# Differentiation of psychotic and affective disorder patients from healthy controls using the niacin skin flush test: a novel analytical method and the SKINREMS system—preliminary research

**DOI:** 10.1038/s41598-026-42991-1

**Published:** 2026-03-08

**Authors:** Ryszard Sitarz, Joanna Rog, Robert Karpiński, Anna Machrowska, Kaja Karakuła, Jacek Bogucki, Dariusz Juchnowicz, Anna Rymuszka, Anna Sierosławska, Hanna Karakuła-Juchnowicz

**Affiliations:** 1 1st Department of Psychiatry, Psychotherapy and Early Intervention, Medical University of Lublin, Gluska Street 1, 20-439 Lublin, Poland; 2 Department of Basic Medical Sciences, The John Paul II Catholic University of Lublin, Lublin, Poland; 3 Department of Machine Design and Mechatronics, Faculty of Mechanical Engineering, Lublin University of Technology, Nadbystrzycka 36, 20-618 Lublin, Poland; 4 Department of Clinical Neuropsychiatry, Faculty of Medicine, Medical University of Lublin, Gluska Street 1, 20-439 Lublin, Poland; 5 John Paul II Catholic University of Lublin, Faculty of Medicine, Lublin, Poland; 6 Department of Psychiatry and Psychiatric Nursing, Medical University of Lublin, Lublin, Poland; 7 Department of Animal Physiology and Toxicology, Faculty of Medicine, The John Paul II Catholic University of Lublin, Lublin, Poland

**Keywords:** Psychosis, Niacin test, Schizophrenia, Schizoaffective disorder, Bipolar affective disorder, Fatty acids metabolism, Biomarkers, Biomarkers, Diseases, Medical research, Neuroscience

## Abstract

According to the phospholipid membrane theory of psychotic disorders, fatty acid metabolism may play a role in the etiopathogenesis of schizophrenia (SCH) and related disorders. Weakened skin reactions to niacin have been documented in SCH and bipolar disorder (BD), prompting research into biomarkers underlying these responses. So far, there is also no standard for performing and interpreting the niacin skin flush test (NSFT) which creates an opportunity for development in this area. The NSFT was conducted based on the SKINREMS system and an original method of analyzing measurements. 120 individuals including 33 patients diagnosed with first-episode psychosis (FEP), 22 with BD, 13 with schizoaffective disorder (SA), 13 with chronic schizophrenia (CS), and 39 healthy controls (HC) participated in the study. NSFT differentiated patients from HC and among subgroups of patients based on color saturation, specific time points, and niacin solution concentrations. The NSFT is a fast, effective, and cheap method that allows the differentiation of individuals suffering from psychotic disorders from HC. Standardization of the test method may contribute to more precise searches for biomarkers responsible of responses in the NSFT and can support the diagnostic process. The presented study is preliminary and exploratory in nature. Developing a method of using NSFT may contribute to a more precise search for biomarkers responsible for the results and thus to the diagnostic process.

## Introduction

Nicotinic acid, an organic compound, an essential human nutrient, otherwise known as a water-soluble vitamin B, contributes to the production of prostaglandins, thereby causing skin flushing when applied to the skin or taken orally^1^. The reaction was discovered in 1959 by Thomas W. Murrell and William M. Taylor who initiated attempts to delve into its causes by subsequent researchers such as Abram Hoffer, Paul Fiedler, and David Horrobin^2–5^. There is extensive scientific evidence that in psychotic disorders, the skin response to niacin is negligible or significantly weakened^6–8^. There are many assumptions as to what exactly can lead to a weak skin reaction in response to the NSFT, including increased activity of phospholipase A2, altered expression of prostaglandin or niacin receptors, reduced arachidonic acid reserves, impaired phospholipid metabolism or impaired capillary wall motility^9^. Studies using the NSFT test have been conducted not only in the case of SCH, FEP, BD, or major depression, but also in other neuropsychiatric diseases such as autism, social phobia, dyslexia, or Huntington’s disease^10–13^. So far, many studies have been conducted using the NSFT, especially engaging patients suffering from SCH. In the 1970s, the phenomenon of a weakened or absent response to niacin in patients was mainly interpreted as a deficit of prostaglandin synthesis, which entailed the NSFT itself as an objective diagnostic biochemical parameter proposed by David Horrobin^14^. These conclusions provided the basis for the formulation of the membrane phospholipid hypothesis of SCH^15^.

As psychotic disorders include not only SCH but also BD and SA, it is worth noting that SA is used in clinical practice as an intermediate condition with a clinical manifestation of both psychosis and mood instability. Such a smooth division of diagnosis should probably be also reflected in the color spectrum results obtained in the NSFT. Regarding the reactions’ spectrum obtained, there are also studies that examined correlations between the intensity of symptoms and sensitivity to the NSFT test using various clinical scales, such as the PANSS scale, i.e. the study by Smesny et al. confirmed the assumptions regarding the intensity of symptoms and the redness of skin in the NSFT, which is a very interesting direction to develop in the context of the diversity of reactions occurring^16^. Over the years, the NSFT has been performed in many different ways. The interpretation of skin reaction was considered by thermal, optical, as well as blood flow and optical spectroscopy change measurements^17–20^.

Still, no standard has been established for performing the NSFT, which creates a space for development and attempts to standardize the method by various teams of researchers.

In this article, we would like to present preliminary study focused on the differentiation of patients diagnosed with FEP, CS, SA and BD using the SKINREMS system. The system was created by a multidisciplinary team of researchers from the 1st Department of Psychiatry, Psychotherapy and Early Intervention in Lublin and the Lublin University of Technology as a simple, easy-to-use, repeatable, portable, and economical device for performing the NSFT, along with an original method of analyzing the measurements taken. The analysis of the results obtained in the study is based on the C&RT algorithm to enhance differentiation between patients with FEP, CS, SA, and BD, and HC.

## Results

### Results of C&RT analysis between patients with specific DSM-5 diagnoses and the HC

**Fig. 1:**
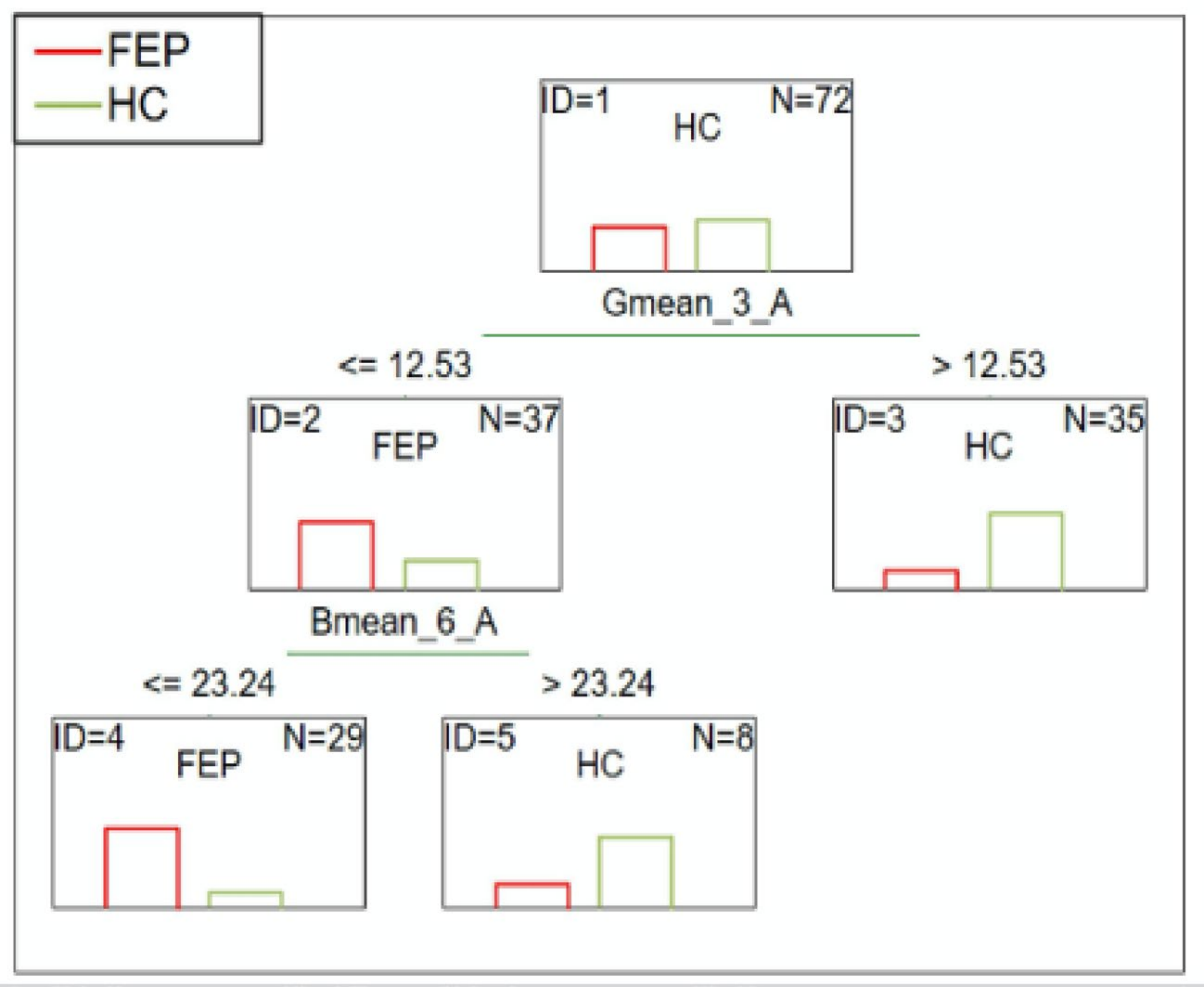
C&RT analysis for FEP and HC, B-blue; G-green; 3-measurement after 2.5-minutes; 6-measurement after 5.5-minutes; A-niacin concentration = 0.1 M.

**Fig. 2:**
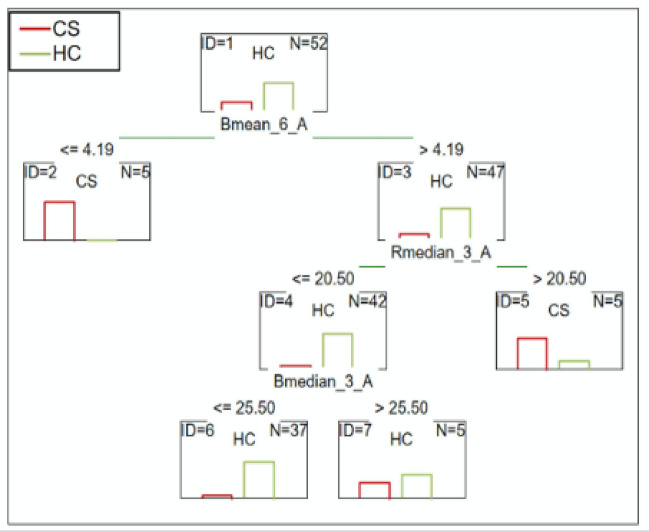
C&RT analysis for CS and HC, B-blue; R-red, 3-measurement after 2.5-minutes; 6-measurement after 5.5-minutes; A-niacin concentration = 0.1 M.

**Fig. 3:**
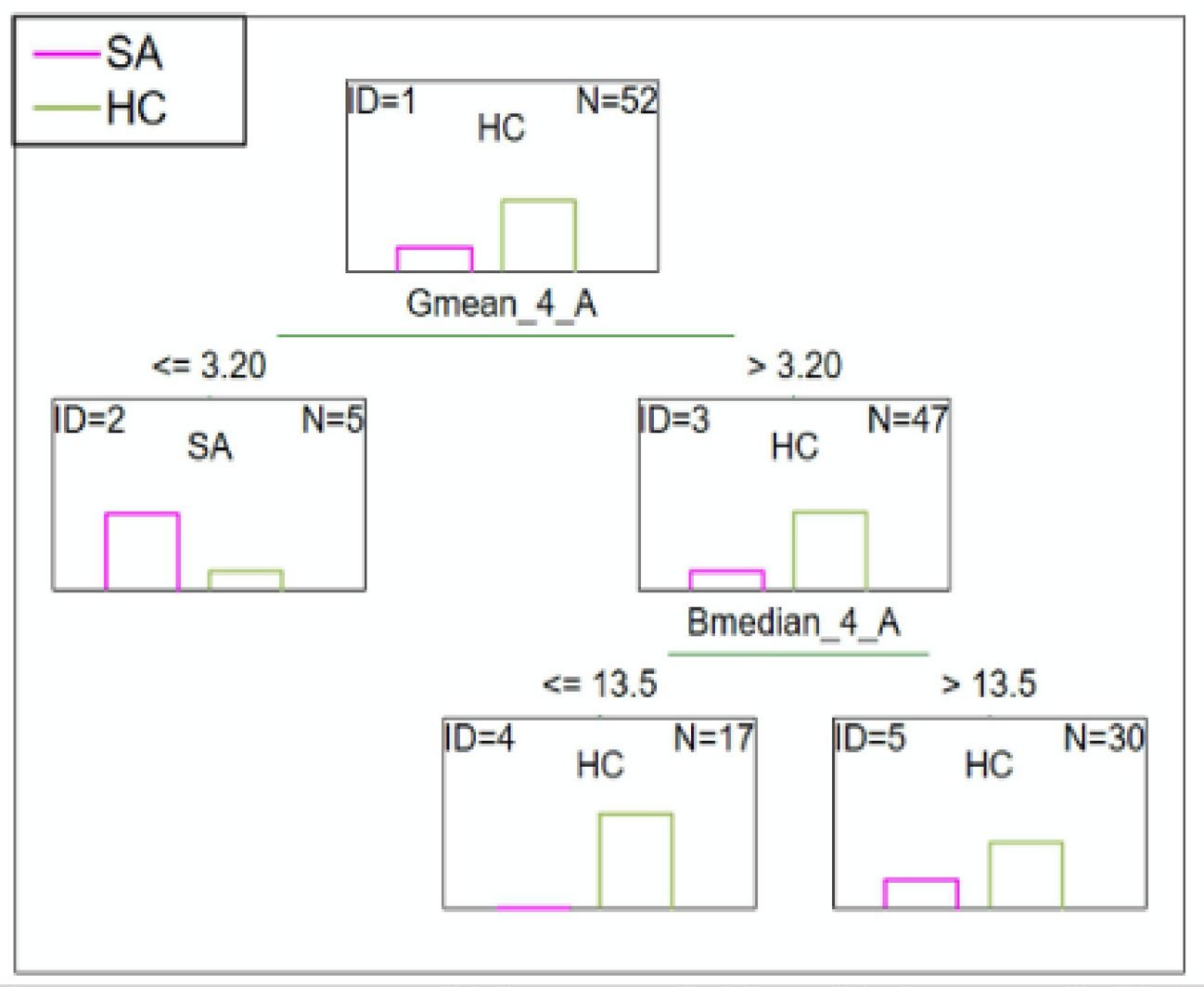
C&RT for SA and HC, G-green; B-blue; 4-measurement after 3.5-minutes; A-niacin concentration = 0.1 M.

**Fig. 4:**
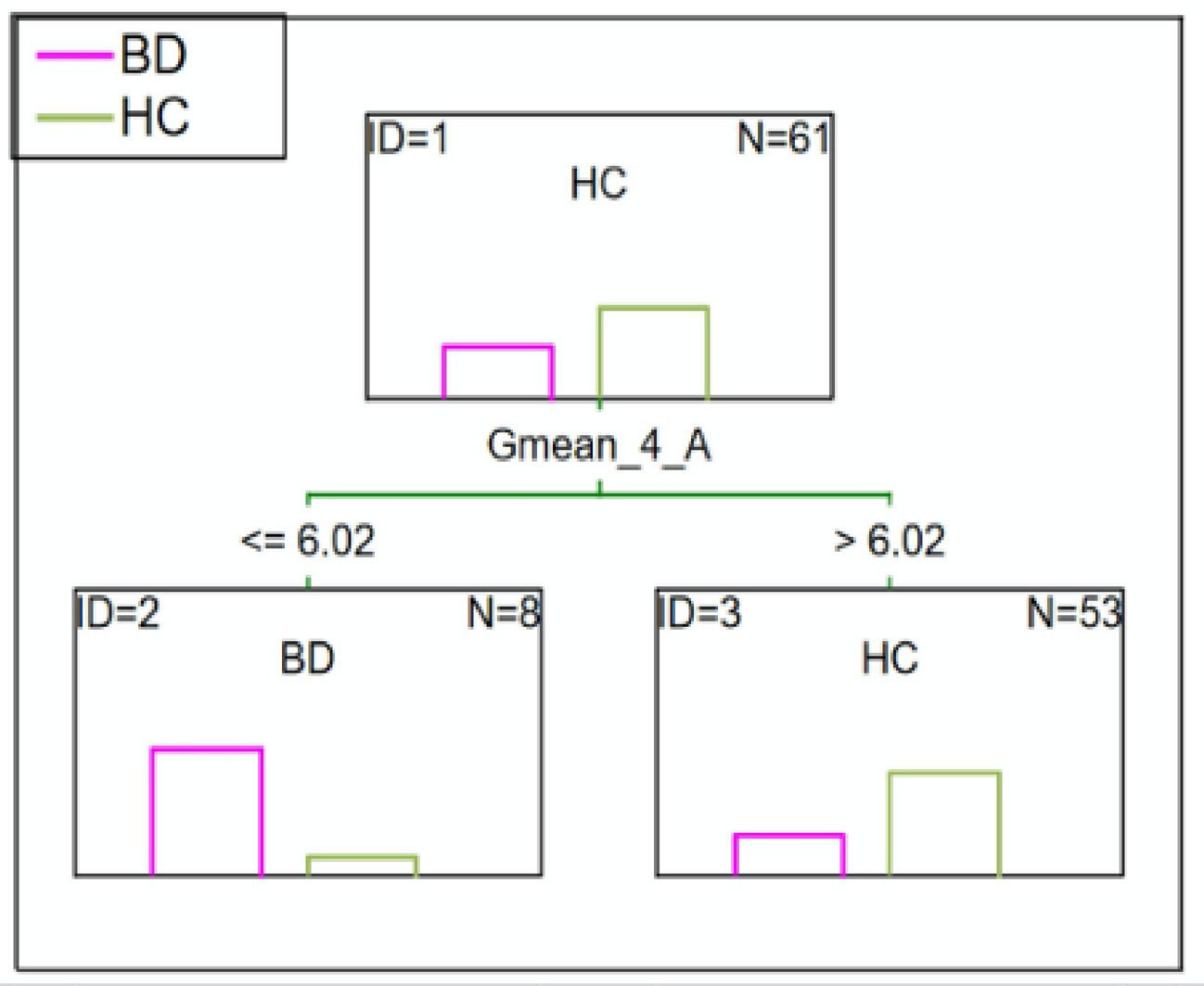
C&RT analysis for BD and HC, G-green; 4-measurement after 3.5-minutes; A-niacin concentration = 0.1 M.

**Fig. 5:**
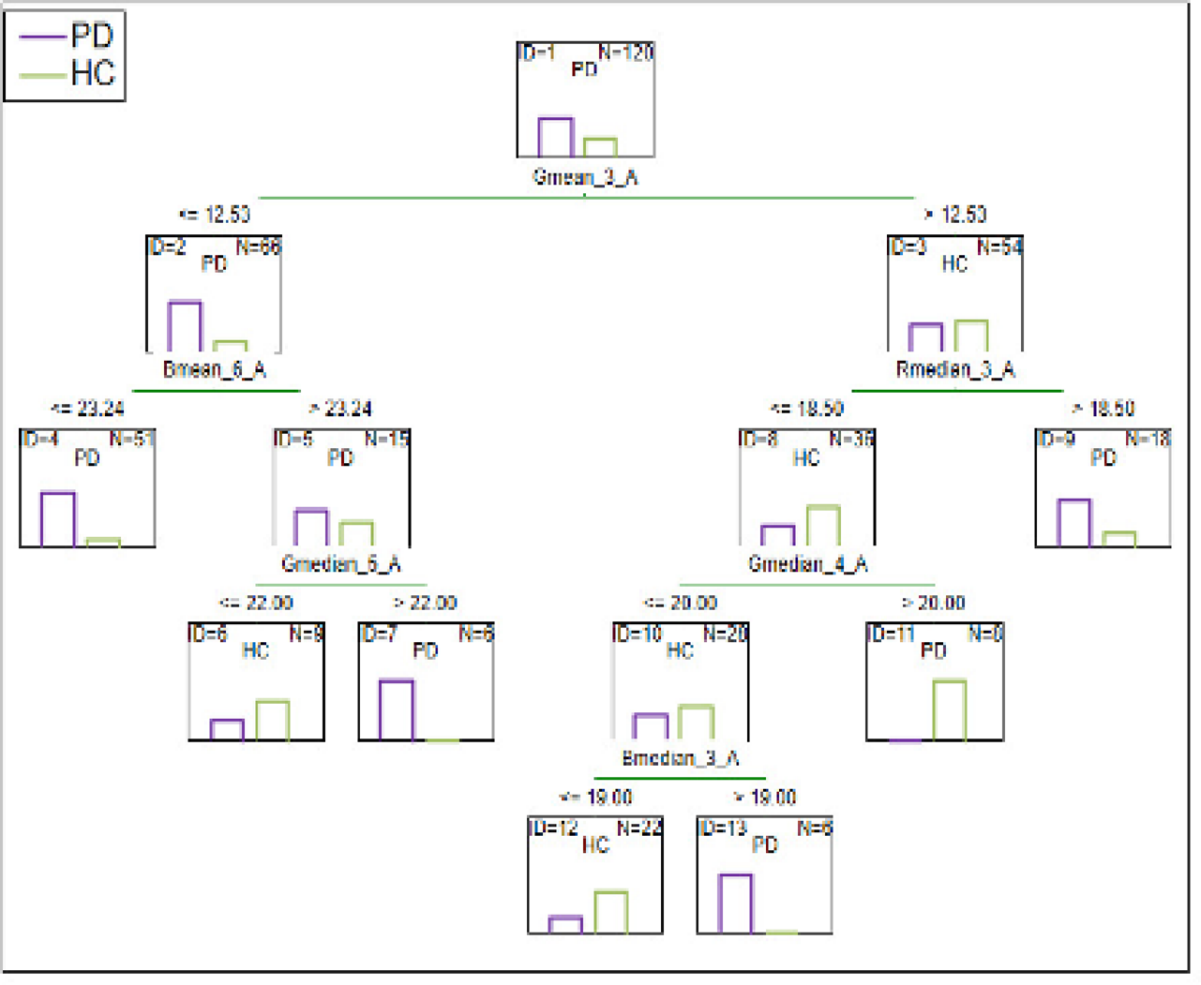
C&RT for the whole group of patients (PD) and HC without disease entities division, G-green; B-blue; R-red; 3-measurement after 2.5-minutes; 4-measurement after 3.5-minutes; 5-measurement after 4.5-minutes; 6-measurement after 5.5-minutes; A-niacin concentration = 0.1 M.

The results of the C&RT analysis are presented in Figs. 1, 2, 3, 4 and 5. We performed analyses to distinguish various patient samples from HC, considering the following groups: FEP, BD, SA, and CS patients. All C&RT analyses performed in the study revealed that the 0.1 M niacin concentration was the group-differentiating factor in each combination. Figure 5 presents a summary tree differentiating the group of patients from HC.

#### FEP patients vs. HC

The C&RT analysis showed two differentiating variables: Gmean_3_A and Bmean_6_A. After 2.5 min, an average green color less than or equal to 12.53 distinguished FEP patients from HC. As the test continued, after 5.5 min, an average blue color less than or equal to 23.24 further separated the FEP sample from HC. The ROC value was 0.80, which suggests a good ability to differentiate these groups (Fig. 1).

#### CS patients vs. HC

The group consisted of CS individuals (more than five years diagnosed with SCH according to DSM-5). C&RT analysis showed three combinations of differentiating variables: Bmean_6_A, Rmedian_3_A, and Bmedian _3_A. After 5.5-minutes the average blue color less than or equal to 4.19 differentiated CS patients from the HC. HC were also differentiated by the median red color equal to or below 20.50 after only 2.5-minutes of performing the test. At the same time, the median blue color value below or equal to 25.50 distinguished the HC from the patients. The ROC value of 0.90 suggests excellent differentiation ability (Fig. 2).

#### SA patients vs. HC

C&RT analysis showed that the mean green color intensity less than or equal to 3.20 after 3.5-minutes distinguished the SA from the HC group. After 3.5-minutes the median blue color less than or equal to 13.5 also discerned the group further. The ROC value was equal to 0.79 suggests a fair differentiation ability (Fig. 3).

#### BD patients vs. HC

C&RT analysis also identified an average green color of less than or equal to 6.02, which differentiated BD patients from the HC after 3.5-minutes. The ROC value was equal to 0.64 suggests a poor differentiation ability (Fig. 4).

#### C&RT analysis summarizing the differentiation between patients and HC without disease entities division

RGB was the model differentiating in the study, as opposed to the HSV, which was also analyzed. The most differentiating were the measurements after 2.5, 3.5, 4.5, and 5.5-minutes. Additionally, measurements within the concentration of 0.1 M were significant, compared to 0.01 M and 0.001 M, which were also performed. The ROC value for differentiating BD patients is equal to 0.88 and suggests a good differentiation ability (Fig. 5).

### Results of C&RT analysis between patients with specific DSM-5 diagnoses

**Fig. 6:**
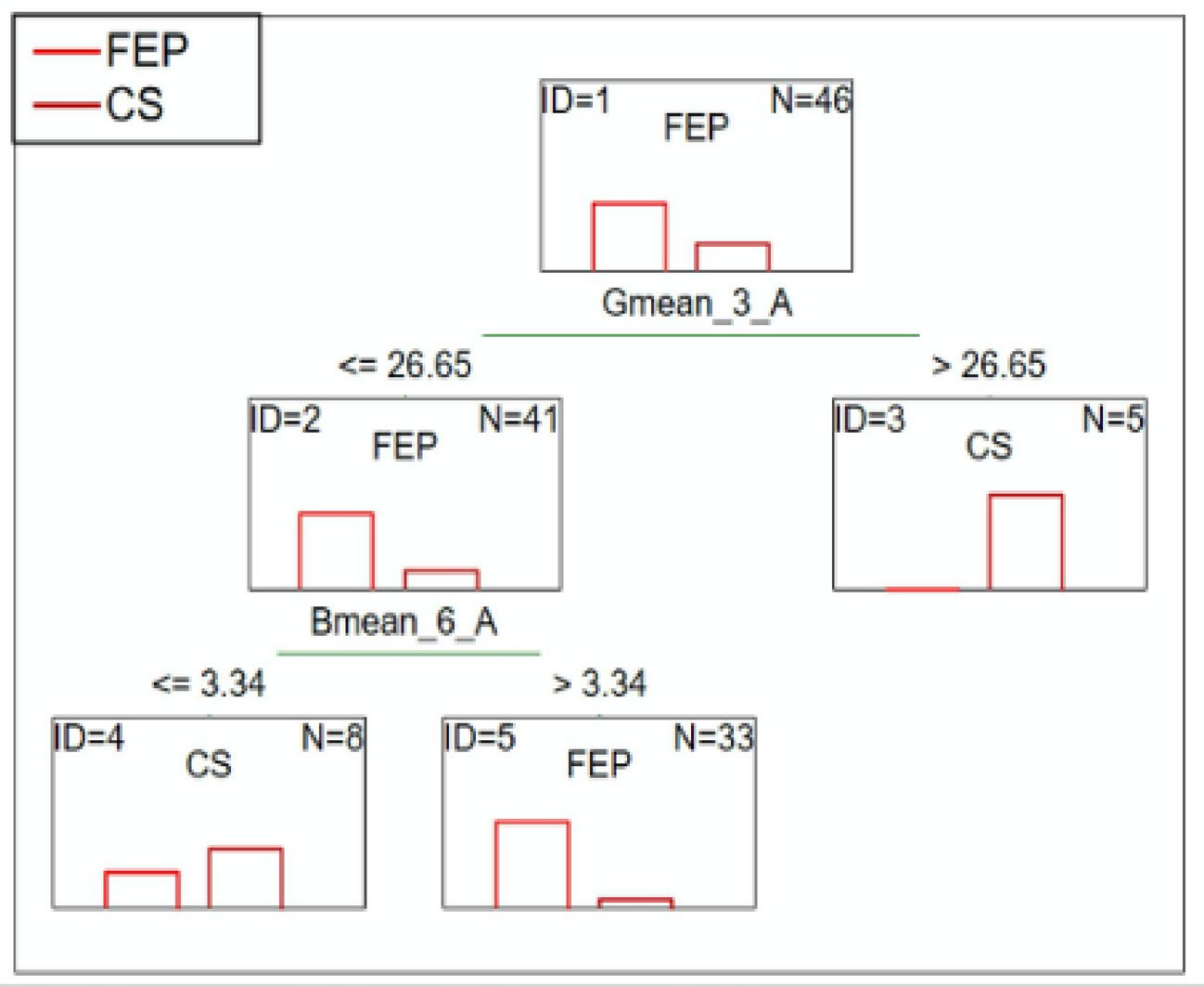
C&RT analysis for FEP and CS patients, B-blue; G-green; 3-measurement after 2.5-minutes; 6-measurement after 5.5-minutes; A-niacin concentration = 0.1 M.

**Fig. 7:**
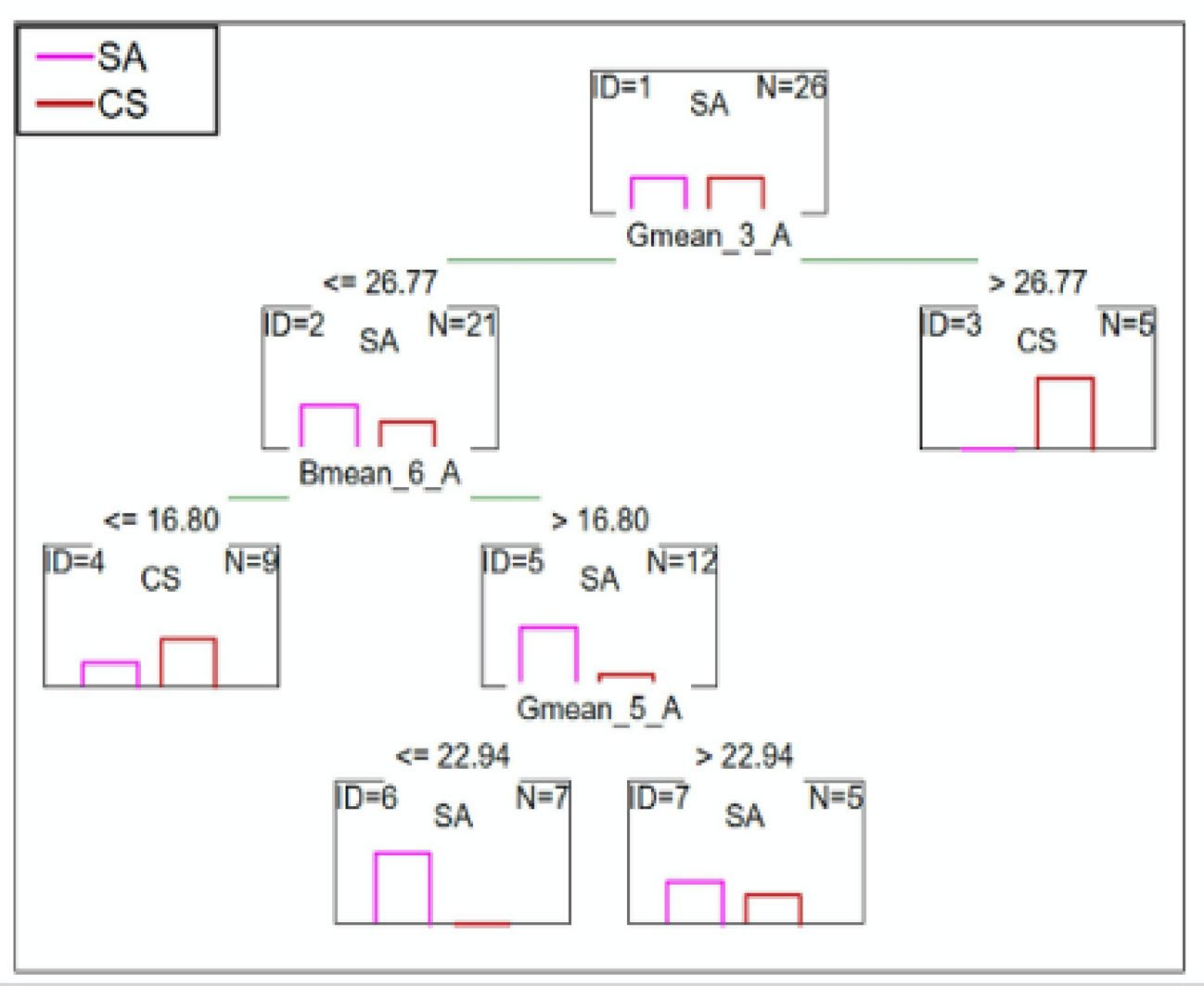
C&RT analysis for SA and CS patients, B-blue; G-green; 3-measurement after 2.5-minutes; 5-measurement after 4.5-minutes; 6-measurement after 5.5-minutes; A-niacin concentration = 0.1 M.

**Fig. 8:**
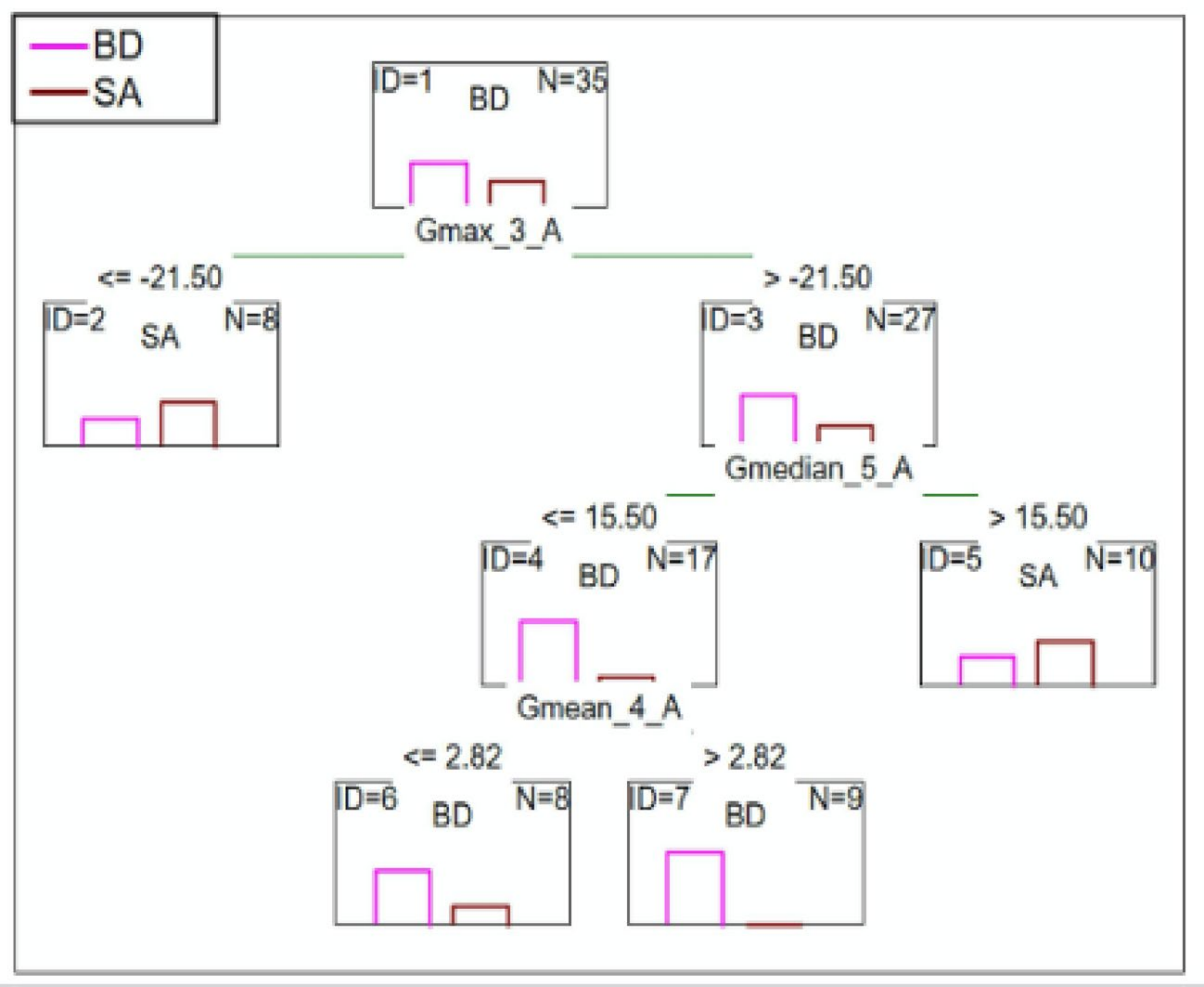
C&RT analysis for BD and SA patients, G-green; 3-measurement after 2.5-minutes; 4-measurement after 3.5-minutes; 5-measurement after 4.5-minutes; A-niacin concentration = 0.1 M.

The results of the C&RT analysis are presented in Figs. 6, 7 and 8. We performed analyses to distinguish various patient samples with specific DSM-5 diagnoses from each other.

#### FEP patients vs. CS patients

C&RT analysis showed that the average green color less than or equal to 26.65 after 2.5-minutes of the test, and the blue color less than or equal to 3.34 after 5.5 min, which differentiated the groups. The ROC value of 0.86 suggests a good differentiation ability (Fig. 6).

#### SA patients vs. CS patients

C&RT analysis showed the mean green saturation of less than or equal to 36.77 after 2.5 min differentiated these two groups. Further, the mean blue saturation of less than or equal to 16.80 and the mean green color less than or equal to 22.94 after 5.5 min also discriminates individuals in this group. The ROC was equal to 0.89 suggest a good differentiation ability (Fig. 7).

#### BD patients vs. SA patients

C&RT analysis showed that after 2.5-minutes, the group was differentiated by the maximum saturation of green color. After 3.5 and later 5.5-minutes patients were differentiated by the mean and median saturation of green color. The ROC value of 0.80 suggests a good differentiation ability (Fig. 8).

Below we present a summary in Table 1 that includes the results discussed above.

**Table 1:** Summary table of the results obtained in the study.

	Color channel	Time point [in minutes]	Concentration of niacin	Cut-off point	ROC-AUC
FEP vs. HC	Green	2.5	0.1 M	12.53	0.80
	Blue	5.5		23.24	
CS vs. HC	Blue	2.5		25.50	0.90
	Red	2.5		20.50	
	Blue	5.5		4.19	
SA vs. HC	Green	3.5		3.20	0.79
	Blue	3.5		13.5	
BD vs. HC	Green	3.5		6.02	0.64
FEP vs. CS	Green	2.5		26.65	0.86
	Blue	5.5		3.34	
SA vs. CS	Green	2.5		36.77	0.89
	Green	4.5		22.94	
	Blue	5.5		16.80	
BD vs. SA	Green	2.5		21.50	0.80
	Green	3.5		2.82	
	Green	4.5		15.50	

## Discussion

The aim of the study was to verify that NSFT based on the SKINREMS method and the author’s original analysis of the results would differentiate the group of patients from the HC. Conclusions should be drawn here regarding the observation of the study methodology itself. Firstly, as regards the concentrations of niacin used in the NSFT, the only differentiating concentration for all comparisons was the concentration of 0.1 M. Secondly, NSFT in our study performance lasted 15-minutes. What is significant, the C&RT analysis showed that the most diagnostic measurements were those lasting from 2.5-minutes to 5.5-minutes of the NSFT. Moreover, in the RGB color analysis, the most differentiating colors were green and blue. Only in the case of CS patients did the saturation of the red color differentiate the group from the HC. Importantly, in each case of differentiating patients from the HC, we must take into account the combination of these three variables, i.e. niacin concentration, time, and color. In our study, no clear influence of the following parameters: age, gender, presence of chronic disease, family history of mental health problems, BMI, physical activity, or tobacco smoking on group stratification in NSFT was observed.

Since there is still no standard for NSFT, there are many reports that show a variety of study results^21,22^. There are also scientific reports about recurrent unipolar depressive disorder and NSFT^23^. Many studies also differ in the time to perform the NSFT^24,25^. Some reports are consistent with our study regarding the most reliable concentration for differentiating patients from HC^26^. Although from 1986, there is also scientific evidence stating that NSFT is not diagnostic for SCH^27^.

The phenomenon in which the green and blue channels demonstrate higher predictive value than the red channel in differentiating erythematous responses in NSFT can be explained by both biological mechanisms and image-analysis methodology. Although erythema is classically associated with an increase in the red component, digital colorimetric analysis enables detection of subtle hemodynamic changes that do not necessarily manifest as intense visible redness.

From a biological perspective, the spectral absorption characteristics of hemoglobin play a key role^28,29^. Oxyhemoglobin (HbO₂) exhibits two principal absorption maxima: in the range of 415–425 nm (blue light) and 540–580 nm (green–yellow light), whereas deoxyhemoglobin (Hb) shows increased absorption around approximately 555 nm and reduced absorption in the blue range^30–33^. Variations in capillary blood oxygenation within the superficial layers of the skin, occurring in response to the inflammatory stimulus induced by niacin, may lead to shifts in light absorption and reflectance that are detected earliest and most distinctly in the green and blue channels. This effect is particularly relevant in the initial phases of the reaction, when microvascular alterations and oxygenation shifts are not yet visible to the naked eye as erythema. Moreover, red light (with a longer wavelength) penetrates deeper skin layers and undergoes greater scattering, resulting in lower contrast between the reaction area and surrounding tissue, as well as a higher risk of digital signal saturation in cases of strong inflammatory responses^34^.

From a methodological standpoint, the green and blue channels provide a broader dynamic range and higher sensitivity to minor changes in blood perfusion and oxygenation within superficial skin layers. In the red channel, an intense vascular response may lead to digital signal saturation, thereby limiting the ability to differentiate between response levels, which was clearly observed during the experiment. At the same time, temporal variability of green and blue channel intensities enables capture of earlier and more subtle stages of the skin response that may have diagnostic relevance before the development of fully expressed erythema. The application of differential color analysis relative to control regions (skin not exposed to niacin) reduced the influence of individual pigmentation and lighting conditions, further improving the precision of change detection in these channels. These mechanisms confirm that the green and blue channels provide more informative signals regarding the vascular and metabolic response of the skin than the red channel, particularly in the context of early and moderate reactions. Consequently, their higher predictive value in NSFT analysis is not inconsistent with the physiology of erythema, but rather complements it by highlighting the need to consider the full spectrum of colorimetric changes in the assessment of skin responses to niacin. Transformation into the HSV color space is nonlinear and results in mixing of information from the three RGB channels. Parameters such as Hue become unstable at low color saturation, while Saturation and Value do not clearly separate the contributions of hemoglobin and melanin. As a result, the direct RGB channels retain a stronger relationship with the physical processes of light absorption by blood, which explains their greater predictive utility compared with HSV parameters^35–37^. The variability in NSFT outcomes across psychiatric diagnoses highlights its potential to reveal pathophysiological mechanisms underlying specific disorders. Our findings highlight the importance of standardizing NSFT protocols, as the differentiation ability of the test was significantly influenced by the concentration of niacin used and the timing of the measurements, which could account for discrepancies observed in previous studies. Table 2 below presents an overview of studies using NSFT, arranged chronologically with the most important observations from the studies conducted.

**Table 2:** Studies conducted using NSFT on different groups of patients in chronological order; SCH - schizophrenia, BD - bipolar disorder, HCs - healthy controls, UD - unipolar depression, MDD - major depressive disorder, AD - affective disorder, GSZ - general schizophrenia patients, VOSZ - violent offenders with schizophrenia.

	Study	Year of publication	Individuals	Country	Observations from the study
1.	Horrobin^5^	1980	*N*/A	UK	In 1980, David Horrobin concluded from his observations that most patients suffering from SCH did not show skin flushing after oral ingestion of 250 mg of niacin, as did the control group.
2.	Rybakowski et al.^17^	1991	33 SCH 18 AD	Poland	In 1991, J. Rybakowski and R. Weterle demonstrated that after oral niacin administration to patients suffering from depression and those with SCH, the subgroup suffering from SCH did not exhibit skin flushing, while patients diagnosed with depression did exhibit noticeable skin redness.
3.	Glen et al.^18^	1996	126 SCH	UK	In the study, patients took 200 mg of niacin orally and were then observed for changes in skin redness for one hour. Importantly, the study took into account plasma fatty acids in relation to skin reactions. It was shown that arachidonic acid levels had the greatest impact on skin reactions.
4.	Hudson et al.^9^	1997	33 SCH 18 BD 28 HCs	Canada	Hudson et al. conducted a study on a group of patients with SCH and BD using a method to measure skin temperature changes following oral administration of 200 mg niacin. 42.9% of patients with SCH showed no response to the niacin test, 6% of patients with diagnosed BD, and 28 HCs showed a normal vasodilation response.
5.	Ward et al.^25^	1998	35 SCH 22 HCs	UK	Ward et al. conducted a niacin test study, applying four different concentrations of niacin to the patients’ forearms and assessing the skin reaction after five minutes. The results were consistent with the concept of reduced arachidonic acid levels in SCH in the context of prostaglandin D2 production.
6.	Hudson et al.^38^	1999	23 SCH 30 HCs	Canada	In the aforementioned study, Hudson et al. demonstrated that significantly more patients diagnosed with SCH were insensitive to the niacin test. Additionally, post-hoc analyses indicated that phospholipase A2 also plays a role in skin reactivity.
7.	Puri et al.^8^	2001	21 SCH 21 HCs	UK	Puri et al. replicated a study in which niacin solutions were applied to patients’ skin and changes were observed over time, giving a sensitivity of the niacin skin test of 90% and a specificity of 75%. The reactions were significantly weaker in patients diagnosed with SCH.
8.	Puri et al.^39^	2002	27 SCH 26 HCs	UK	In the study by Puri et al., a volumetric index called the volumetric niacin response was determined, based on which a threshold level was established that differentiated individuals diagnosed with SCH from the HCs with a sensitivity of 78% and a specificity of 65%.
9.	Smesny et al.^19^	2003	25 SCH 25 HCs	Australia	In the Smesny et al. study, the skin response to niacin was assessed using optical reflection spectroscopy in patients with first epilepsy-psychosis. The analyses took into account variables such as skin response and its time course, skin color assessment, and edema. The response was shown to be weaker in the patient group than in the HCs, allowing for the identification of individuals with metabolic impairment.
10.	Messamore et al.^7^	2003	27 SCH 21 HCs	USA	In the study, the niacin test was assessed using laser Doppler flowmetry. The results indicate that in patients diagnosed with SCH, an impaired response to the niacin test is associated with abnormalities in signal transduction mechanisms, receptors, and enzyme activity that could influence the release, synthesis, and/or response to prostaglandins.
11.	Tavares et al.^40^	2003	38 SCH 28 HCs	Brazil	In 2003, Tavares et al. focused on examining phospholipase A2 activity in patients diagnosed with SCH who underwent the niacin test. They found that phospholipase A2 activity was higher in patients diagnosed with SCH. Furthermore, the highest activity was observed in patients who, across the spectrum of reactions, showed no reaction at all. Furthermore, after eight weeks of treatment with antipsychotic drugs, phospholipase A2 activity was observed to be reduced.
12.	Ross et al.^20^	2004	27 SCH 26 BD	Canada	In a study by Ross et al., skin reactions were compared in patients diagnosed with SCH and those with BD using laser Doppler flowmetry. The study found weaker reactions in patients with SCH compared to individuals diagnosed with BD and HCs.
13.	Lin et al.^41^	2006	153 SCH 94 HCs	Taiwan	Lin et al. undertook to examine not only patients but also their healthy family members for their response to the NSFT. Liu et al. tested patients, parents, siblings, and a control group using three concentrations of niacin applied to the forearm. However, this method is not equivalent to genetic testing. There are also many variables that could have influenced the final results.
14.	Liu et al.^21^	2007	61 SCH 18 BD	Taiwan	In the aforementioned study, the niacin test was performed on patients diagnosed with SCH, bipolar mania, and HCs. A significantly weaker niacin test response was demonstrated in patients with SCH, while a similar response was observed in bipolar mania and HCs. The niacin test demonstrated 49.2% sensitivity and 92.5% specificity in individuals with SCH compared to healthy controls. The results were independent of smoking status.
15.	Yao et al.^42^	2015	70 SCH 59 BD	USA	In a study by Yao et al., a laser Doppler flowmeter was used to measure the niacin test result. The concentration of niacin required to induce half-maximal blood flow was determined, and the results were assessed based on this parameter. Patients diagnosed with SCH had the weakest skin reactions. Furthermore, factors such as race, age, smoking history, or gender did not influence the results.
16.	Sun et al.^43^	2017	163 SCH 63 MDD 63 HCs	China	A study by Sun et al. confirmed that the niacin test can distinguish patients with SCH from those with affective disorders and HCs. According to the researchers, the weakened response to niacin may reflect impairment in membrane fatty acid composition, as well as abnormalities in phospholipase 2 A function, lipid metabolism, and oxidative stress.
17.	Karakula-Juchnowicz et al.^44^	2020	56 SCH 29 BD 45 HCs	Poland	Karakula-Juchnowicz et al. in their study showed 91% sensitivity and 72% specificity in distinguishing individuals with SCH from individuals with BD, and 71% sensitivity and 66% specificity in discriminating individuals with SCH from HCs after conducting the niacin test using their method.
18.	Wang et al.^6^	2021	307 SCH 179 BD 127 UD 148 HCs	China	A study by Wang et al. found that patients with SCH, depression, and BD exhibited similar responses to niacin. A bivariate cut-off was established, distinguishing patients diagnosed with SCH from those with affective disorders. According to the authors, the obtained results broaden the perspective on common biological basis and shared pathophysiology. Data such as age, BMI, gender, smoking habits, educational status, and alcohol dependence were taken into account in the analysis.
19.	Hu et al.^45^	2022	82 SCH 41 BD 80 HCs	China	The study was conducted by Hu et al. using a laser Doppler flowmeter. Furthermore, cut-off bivariates and a quantitative indicator called the overall trend area were established, based on which the results were analyzed. The authors of this interpretation method state that with its use, the NSFT can be a useful tool dedicated to patients with FEP.
20.	Zhang et al.^46^	2023	60 CHR 60 HCs	China	Zhang et al. conducted a NSFT, taking into account markers of inflammation and individuals identified as being at clinically high risk of psychosis. Conversion to psychosis within two years was considered based on niacin test results and inflammatory imbalances. Levels of IL-1β, IL-2, IL-6, IL-8, IL-10, and tumor necrosis factor-α were examined, and NSFT results were interpreted using laser Doppler flowmetry. The study results confirm a significant association between niacin test response and conversion to psychosis. Interestingly, none of the cytokine levels were significant.
21.	Ju at al.^47^	2025	54 FEP 52 HCs	China	Ju et al. conducted a study focusing on abnormal responses to the NSFT in FEP patients, with an emphasis on assessing cognitive impairments in these patients. Patients with the weakest niacin test responses demonstrated the greatest severity of negative symptoms and higher PANSS scores. Correlation revealed a significant positive relationship between overall symptom severity and niacin skin reaction.
22.	Wang et al.^48^	2025	269 SCH	China	In a study from 2025, Wang et al. administered a niacin test along with the Positive and Negative Syndrome Scale, a repeatable battery for the assessment of neuropsychological status, insight, and treatment attitudes questionnaires, to individuals diagnosed with schizophrenia, divided into two subgroups: treatment-resistant SCH and non-treatment-resistant SCH. The study showed that the niacin test has poor efficacy in recognizing treatment-resistant SCH.
23.	Lyu et al.^49^	2025	120 cohort of patients and healthy individuals	China	This study demonstrates the use of the NSFT in artificial intelligence analysis. Skin lesions from patients with SCH, BD, and unipolar depression were considered. The results show that the machine learning diagnostic method achieves sensitivity results between 60.0 and 65.0% and specificity between 75.0 and 88.3% when considering the above-mentioned diagnoses.
24.	Chen et al.^50^	2025	315 VOSZ 296 GSZ 281 HCs	China	Chen et al. conducted a niacin test on a group of patients with SCH, dividing them into those with a history of violence and those without such a history. There was no significant difference in variables such as sex, BMI, or age between the two groups. Both groups of patients with SCH showed similar response results to the NSFT compared to the HCs. Furthermore, factors such as antipsychotic medication dosage, severity, or violence were not significant. Therefore, the NSFT demonstrated its usefulness in distinguishing patients from HCs, but it did not differentiate patients with a history of violent crimes.

## Limitations

There are many variables that can contribute to the wide range of different skin reactions in the NSFT. It can be influenced by skin and ambient temperature, humidity, proximity of vessels in the ​​application area of the niacin solution, hormonal status, niacin concentration, length of the test, and method of evaluation.

Regarding the limitations of our method, there are issues that could be improved. The first one is the application of niacin solutions, which were performed manually. It can be expected that the test time for every niacin solution patch could be somehow shifted. Automating the method of applying solutions at the same time would contribute to increasing the precision of the test.

Considering the individual limb structures, the application distances between the niacin patches should be organized the same. On the other hand, the study showed that the only best-differentiating concentration of niacin solution is equal to 0.1 M.

We used C&RT analysis to predict the NSFT variables linked to psychiatric diagnosis. The C&RT disadvantages should be taken into consideration in the interpretation of obtained results. This data mining method generates binary trees and cannot handle multiclass problems. We analyzed populations with different diagnoses, and multi-analysis could better characterize the examined population^51^. However, considering the common psychopathology of examined diseases, multiclass analysis could be confusing and flatten the problem of diagnostic similarities. Overfitting is another issue in C&RT analysis. The most used solution is preparing by setting a criterion during the tree growth process^51^. We limited the variables input in the model by the initial selection and the elimination parameters dependent on clinical or sociodemographic factors. Another limitation of this study is that although sociodemographic, lifestyle, and health-related variables were systematically examined and excluded in the algorithm proposed by us, their potential indirect effects on NSFT-based group stratification and the presented results cannot be completely ruled out. This study should be interpreted as hypothesis-generating, and in future studies performed on larger cohorts, preferably from different countries or geographical areas, formal internal validation of the models will be needed. The relatively small number of participants in the study may have influenced the statistical power; therefore, the results cannot be generalized to larger populations, as the study’s character is preliminary and experimental.

## Conclusions

To conclude, the NSFT shows great promise as a practical diagnostic tool for distinguishing individuals with FEP, SCH, SA, and BD from healthy controls. This simple test, which assesses fatty acid content in cell membranes, could also play a role in personalizing treatment strategies, particularly through tailored dietary recommendations. For instance, supplementing polyunsaturated fatty acids might help delay the onset or slow the progression of these disorders, even in their early stages. Improving the methodology of the NSFT and exploring the factors that influence its outcomes could expand its utility, not only in diagnostics but also in developing therapeutic and preventive strategies. Moreover, the test’s ability to reveal insights into the underlying psychopathology of specific psychiatric disorders highlights its broader potential in both clinical practice and research. However, to fully realize its capabilities, it is essential to establish a validated and standardized protocol for conducting the NSFT and interpreting its results reliably.

## Materials and methods

### Characteristics of study participants

The study group consisted of 120 individuals: 33 FEP, 22 BD, 13 SA, 13 CS patients, and 39 HC. Among patients, there were 41 females (50.62%), 24 cigarette smokers (29.63%), and 18 individuals diagnosed with additional somatic diseases (22.22%). A family history of mental health conditions was declared by 20 individuals (24.69%). The median age in the group was 27 years (15–56 years). The statistical difference at the level of *p* = 0.002 was demonstrated by body mass index (BMI) variable, among patients the median BMI was 24.58 kg/m^2^, while the HC group showed a BMI of 21.31 kg/m^2^. Another statistically significant difference was in level of physical activity *p* = 0.005, expressed in minutes per week. In the patient’s group, it was 0 min, while in the study group, the median was 45 min per week. Additionally, the median duration of illness for patients in years was 5, the median number of hospitalizations was 2, the olanzapine equivalent in relation to the used antipsychotic drugs 25 mg, and the median score on the Positive and Negative Syndrome Scale (PANSS) scale was 74 points (Table 3)^52^.

**Table 3:** Sociodemographic data of the study participants.

	Patients	Healthy individuals	Differences
*N* (%)			
Gender [females]	41 (50.62)	25 (64.10)	0.164
Cigarettes [smokers]	24 (29.63)	7 (17.95)	0.180
Somatic conditions [yes]	18 (22.22)	11 (28.20)	0.473
Mental health conditions [presence in family]	20 (24.69)	4 (10.26)	0.064
Median (Min-Max)			
Age [years]	27 (15–56)	25 (23–32)	0.379
BMI [kg/m^2^]	24.58 (17.99–38.75)	21.31 (17.71–29.12)	0.002
Physical activity [min/week]	0 (0-1440)	45 (0-630)	0.005
Duration of illness [years]	5 (1–31)	N/A	N/A
Hospitalizations [number]	2 (0–15)	N/A	N/A
Olanzapine equivalents [to 1 mg OLA]	25 (0.63–135)	N/A	N/A
PANSS [total points]	74 [36–145]	N/A	N/A

### Criteria for the study

Inclusion criteria for patients:

(1) Informed written consent, (2) Women and men aged 18–50, (3) Diagnosis of SCH, BD with psychosis, or SA according to DSM-5 criteria.

Inclusion criteria for HC:

(1) Informed written consent, (2) Women and men aged 18–50.

Exclusion criteria for patients:

1. Lack of consent.
2. The occurrence of diseases that may affect vascular tone, skin diseases, autoimmune, cancer, cardiovascular diseases, and other somatic in an unstable phase or active inflammation.
3. Current use of antibiotics, lipid-lowering drugs, antihistamines, anti-inflammatory drugs, and drugs that change the calcium metabolism.
4. Use of supplements such as unsaturated fatty acids, particularly omega-3 fatty acids within the three months before the study.
5. Use of vitamins or a dietary supplement containing niacin dose above 100 mg/day or the use of any dose of cannabidiol.
6. Allergies, including known allergies to the test compound.
7. Organic damage to the nervous system.
8. Addiction other than to caffeine and/or nicotine.
9. The occurrence of major mental disorders other than SCH, SA, and BD according to DSM-5.
10. Pregnancy or breastfeeding.

Exclusion criteria for the HC:

1. Lack of consent.
2. The occurrence of diseases that may affect vascular tone, skin diseases, autoimmune, cancer, cardiovascular diseases, and other somatic diseases in an unstable phase or active inflammation.
3. Current use of antibiotics, lipid-lowering drugs, antihistamines, anti-inflammatory drugs, and drugs that change the calcium metabolism.
4. Use of supplements such as omega-3 fatty acids within the three months before study.
5. Use of vitamins or a dietary supplement containing niacin dose above 100 mg/day or the use of any dose of cannabidiol.
6. Allergies, including known allergy to the test compound.
7. Organic damage to the nervous system.
8. Addiction other than to caffeine and/or nicotine.
9. The occurrence of SCH, SCHAD, BD or other major mental disorders according to DSM-5.
10. The prevalence of mental illness in the family.
11. Pregnancy or breastfeeding.

### Measurement of skin reactions using niacin

The measurements were conducted under the same clinical laboratory conditions. The study used a niacin solution applied to the forearm skin in three concentrations: 0.1 M, 0.01 M, and 0.001 M. Tissue papers (2 × 2-cm) soaked in the niacin solution were in contact with the skin for 90 s. Then, the measuring device recorded the reactions on the skin.

To ensure strict standardization of lighting and limb positioning, the SKINREMS device functions as a closed measurement chamber that isolates the measurement field from external environmental variability. The forearm is inserted through a dedicated opening and positioned on a fixed internal support, which stabilizes limb orientation and maintains a constant camera-to-skin distance across all examinations. This mechanical constraint minimizes variability related to posture, rotation, and viewing angle. The construction is based on a rectangular frame made of aluminum profiles. The system walls are made of polymethyl methacrylate impermeable to light. The device has holes for the camera and the limb. Illumination is provided by an integrated LED light source mounted in a fixed position inside the enclosure. The light spectrum and intensity remain constant during all measurements, and the polymethyl methacrylate walls block ambient light, eliminating fluctuations caused by daylight or room lighting. As a result, all images are acquired under reproducible photometric conditions, which is critical for reliable quantitative color analysis. Camera exposure parameters (focus, exposure time, ISO, and white balance) were fixed within the acquisition application and remained unchanged throughout each session. The lamp used has a convenient power supply from the USB socket al.lowing mobility. The system enables standardized and repeatable measurements. Image acquisition was performed using a 64 MPx (Redmi Note 9 Pro mobile phone) camera at equal time intervals, with a dedicated application controlling the acquisition process. The applied design and procedural solutions enabled the collection of fully repeatable and comparable data, providing a reliable basis for assessing the response to NSFT. A detailed description of the measurement system with diagrams was shown in the previous review paper published by the team^44,53^.

**Fig. 9:**
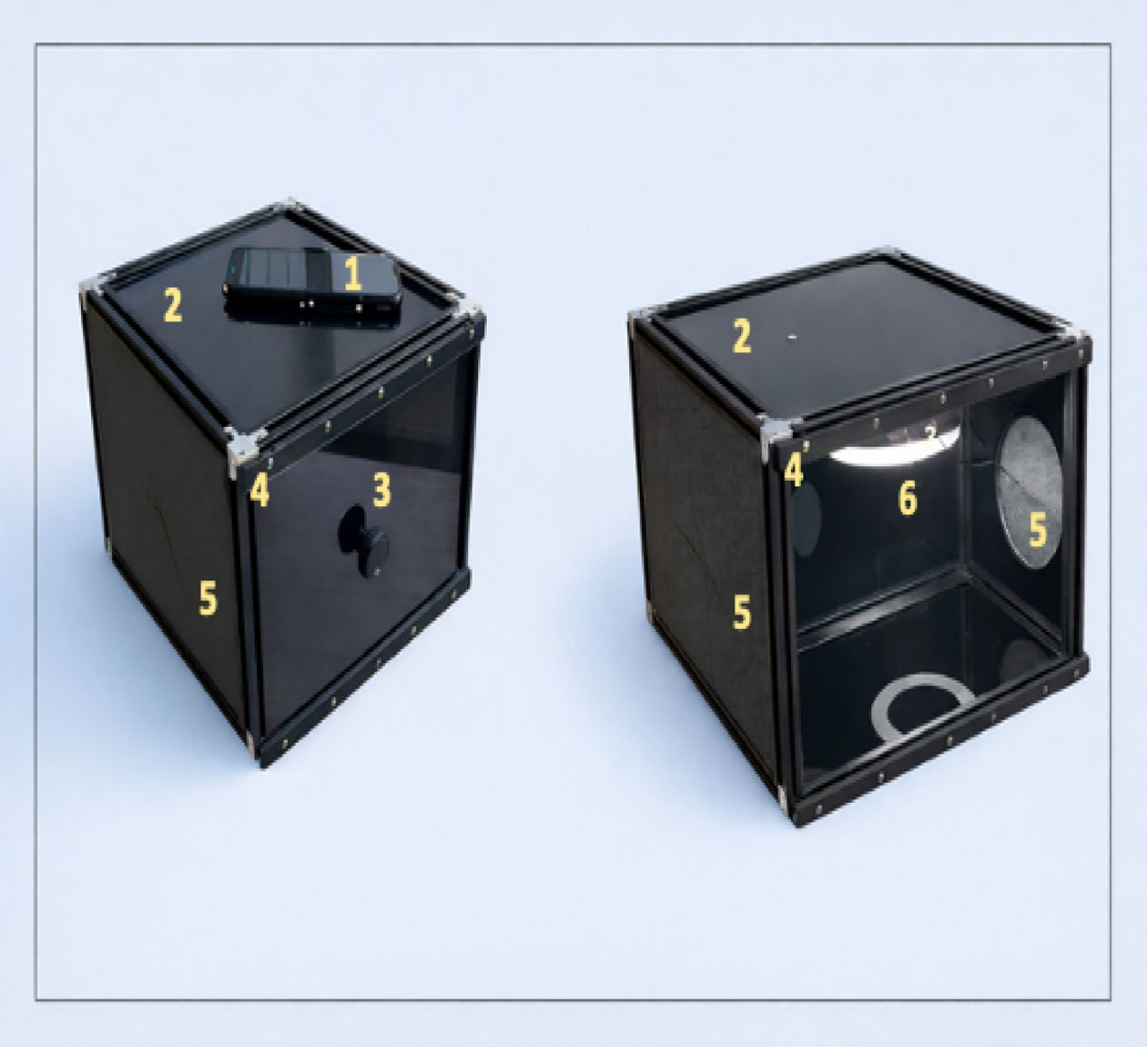
Schematic representation of the SKINREMS device showing the conceptual design of the measurement chamber, illumination system, camera positioning, and subject stabilization developed by an interdisciplinary research team. 1 - Camera, 2 - Plexiglass plate, 3 - Sliding wall handle, 4 - Aluminum profile, 5 -Foam cut-out wall for forearm placement, 6 - Ring lamp.

Analyzing skin changes required image processing algorithms and began with the video recording video for 15-minutes. Time-lapse videos included the propagation of skin changes. The photos were triggered automatically every 60 s. For each individual 15 images for every niacin concentration were obtained during one examination. The observed formation, disappearance, and intensity of the reaction changed over time. The video files were divided into individual frames, which were the subject of further image analysis. The acquired data were subjected to processing based on parameters that may contain diagnostically significant information. A schematic representation of the SKINREMS measurement system is shown in Fig. 9, which presents the first conceptual design of the device developed by an interdisciplinary research team.

### Image processing and skin color model description

Image processing was conducted using a semi-automatic MATLAB-based pipeline. First, frames were extracted at fixed 60-second intervals. Next, regions of interest (ROIs) corresponding to each niacin application site and adjacent control skin areas were defined. ROI boundaries were refined using edge detection and morphological operations to reduce background influence. For each ROI and time point, statistical descriptors of RGB and HSV channels (mean and median) were computed. Final diagnostic variables were expressed as differences between post-niacin ROIs and baseline control skin ROIs from the same frame, thereby reducing the influence of individual skin tone and residual lighting inhomogeneity. The Image Processing Procedure is shown in Table 4.

**Table 4:** Summary of the image processing procedure, including sequential processing steps, applied operations, and extracted quantitative features.

Step		Description
1	Input data preparation	Sequential image frames acquired at predefined experimental time points were subjected to analysis. Each frame was treated as an independent sample for further processing.
2	Definition of regions of interest (ROIs)	For each frame, regions of interest were defined to include the skin area exposed to niacin and a control skin area not exhibiting a reaction. When necessary, background regions were also identified to allow their exclusion from further analysis.
3	Determination of baseline parameters	Individual baseline skin color parameters were established based on the control skin regions. These baseline values served as a reference for subsequent calculations.
4	Skin area segmentation	The regions designated for analysis were segmented using edge-based and texture-based filtering techniques. To improve the quality of segmentation masks, morphological operations including dilation, erosion, opening, and closing were applied, enabling effective separation of the ROIs from the background.
5	Manual correction of segmentation	Due to natural variability in skin coloration and the possibility of weak or visually subtle erythema, manual correction of the ROI boundaries was performed in ambiguous cases.
6	Preservation of original color information	Image analysis was conducted using the original color data without reducing the images to a single intensity channel, in order to preserve the full spectral information describing the skin response.
7	Extraction of color space components	From the segmented ROIs, color components of the RGB space (R, G, B) were extracted. Subsequently, the images were converted to the HSV color space to obtain the H, S, and V parameters.
8	Calculation of statistical descriptors	For each color channel within the ROI and for each time point, a set of statistical measures describing the distribution of pixel intensities was calculated, including the mean, median, mode, minimum, maximum, and range.
9	Normalization with respect to control skin	To reduce the influence of individual differences in baseline skin tone and subtle variations in lighting conditions, differential features were calculated as the difference between values obtained in the reaction area and the corresponding values in the control skin area within the same frame.
10	Feature aggregation and data export	All calculated parameters were compiled into a structured feature table including RGB and HSV components, statistical descriptors, and time points. The resulting dataset was exported to a spreadsheet file for further statistical analysis and model development.

Image processing involved isolating ROIs changed by the niacin from areas with no solution, and background areas. The first step was to determine the baseline parameters for each individual patient. In the next step, image parameters within individual changes related to the application of the niacin solution were determined. The areas subjected to analysis were separated using edge ​​and texture filtration and morphological operations (closure, opening, erosion, or dilation operations).

The original images were preserved in their original colors to expand the number of parameters describing the appearance and area affected by the skin change. The access to the color variables of the physiological reaction (Red, Green, Blue [RGB] and Hue, Saturation, Value [HSV]) with the possibility of comparison with the skin control areas were obtained. Acquisition of RGB variables allowed for a certain standardization of the reaction types in NSFT through the statistical description of particular intensity color changes and the control areas (determination of the mean and median of intensities in the area of isolated limb).

The determination of skin color parameters was performed using the MATLAB R2023b program and a processing algorithm designed by the authors. This algorithm allowed viewing images, selecting areas for analysis, and automatically determining coefficients characterizing skin color. Due to the high sensitivity of the digital camera, even despite the use of a proprietary measurement system, the results may be distorted by changes in lighting conditions invisible to the human eye, therefore the differences in the values ​​of the color components between adjacent irritated and non-irritated skin areas appear to be small.

Skin areas were manually marked to account for natural skin color variations and cases of no skin reaction. Skin response was characterized by differences in color component values, expressing the color change of the irritated area. Color component values ​​were vectors in Cartesian RGB and cylindrical HSV spaces. The RGB and HSV models are used to represent colors, but they differ in their basic assumptions and primary applications. Conversion between RGB and HSV color spaces was performed in the MATLAB environment. The conversion was performed similarly as in the Karakuła-Juchnowicz et al.^44^:

$$H=\{\theta B \le G360-\theta B>G \theta=arcos\frac{1}{2}\frac{\left(R-G\right)+\left(R-B\right)}{\sqrt{{\left(R-G\right)}^{2}+\left(R-B\right)\left(G-B\right)}}$$

$$S=1-3\frac{min\left(R,G,B\right)}{R+G+B}$$

$$V=max\left(R,G,B\right)$$

Finally, color characteristics were determined for each ROI. The set of representative values ​​included: mean, median, mode, range, minimum, and maximum values ​​of the R, G, B, H, S, and V components. The results were collected in the Excel spreadsheet. The values ​​in each of the tested color spaces in individual measurements were subtracted from the base values ​​of skin tones not exposed to the niacin solution. This was to highlight the features that distinguish the skin after the application of the solution from its normal shade. The measurement of natural skin color included determining RGB, HSV components, and their indicators for the entire area of ​​the automatically extracted limb. The aim was to extract features of changes without the influence of the individual skin color of the subjects. To avoid ambiguity in the interpretation of color-related variables, it should be emphasized that all reported RGB and HSV values represent dimensionless numerical intensity levels of digital image channels, derived from pixel brightness values within the analyzed regions of interest. These parameters describe the mean or median signal intensity per pixel and do not refer to pixel count, area, or spatial dimensions.

### Statistical analysis

Obtained data were analyzed using Statistica 13.3 (TIBCO Software Inc., Tulsa, USA)  with *p* < 0.05 indicating significant differences. The Shapiro-Wilk test was used to determine the distribution of quantitative data. As most variables had non-normal distributions, the variables were expressed as median and range. The significance of differences between datasets from study groups was defined using the chi-square (for qualitative data), Student’s t-test, and Mann–Whitney U-test (for quantitative data). The correlation between examined parameters was analyzed using Pearson and Spearman’s rank correlation coefficients. A binary partitioning statistical data-mining technique: C&RT algorithm was used to find the NSFT-based model for classifying subgroups of patients based on diagnosis. The diagnostic ability of the proposed models was further tested using receiver operating characteristic curve (ROC) analysis.

#### Establishing a model for the classification of psychiatric disorders

In biological datasets, high variance often occurs in individuals with the same clinical conditions due to their multi-factorial origin. In diseases that involve genetics and environmental factors, explaining the obtained results is challenging. The study uses a five-step approach to classify the psychiatric conditions.

**Step 1**: Screening procedure of potential variables differentiate individuals with psychiatric diseases.

To determine the potential variables that vary between individuals with psychiatric disease and HC, we compare the results obtained between HC and patient groups. According to analysis, 469 from 1184 (1091 with a non-normal distribution) variables differed between examined populations and were used for analysis.

**Step 2**: Exclusion of the variables affected by sociodemographic, lifestyle and health-related factors.

Many studies confirm NSFT exhibits a strong relationship with age, gender, food habits, tobacco and others. We excluded the NSFT data connected with sociodemographic, lifestyle, and health factors to minimize the risk of bias due to confounders. The relationship was assessed for both groups in the order determined by the researchers. The variable was removed if the connection was found in any group, independently from the strength of the relationship. The following parameters were put into the analysis: age, gender, presence of chronic disease, family history of mental health problems, body mass index (BMI), physical activity (minutes per week) and tobacco smoking (Picture 1). After this step, 137 (92 with a non-normal distribution) variables were found to be not affected by the factors mentioned above.

**Picture 1:**
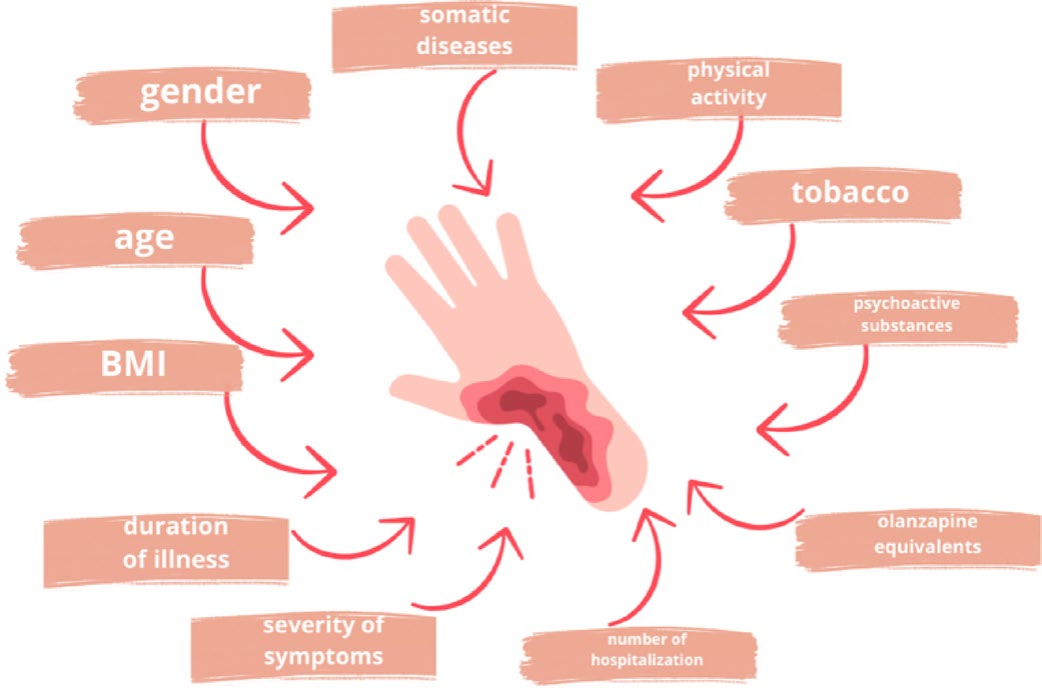
The relationship between NSFT results and sociodemographic, lifestyle and health-related factors.

**Step 3**: Exclusion of the variables affected by the disease-related factors.

This step was performed only in the patient group. The concept that lipid metabolism is engaged in the pathophysiology of psychiatric diseases is well known, and due to potential disturbance, some clinical-related factors may affect NSFT. The ideal diagnostic biomarker should not be affected by drugs or disease severity, progression, or other disease-related factors. We excluded NSFT data connected with disease-related factors such as duration of illness, number of hospitalizations, equivalents of olanzapine, and severity of symptoms. 53 variables have been excluded in this step.

**Step 4**: Machine learning model implementation.

In diagnostic studies, the test should yield binary outcomes, i.e., the presence or absence of the feature. The approach which allows this solution is to determine cut-off points for variables. The continuous values of NSFT were transformed into dichotomous ones (below and upon the cut-off point) using the C&RT method. C&RT allow the identification of features of mutually exclusive populations easily. During analysis, the algorithm generates rules based on which the individuals can be assigned to one of two subpopulations. We established a two-step classification model:

- Presence or absence of psychiatric disease.
- Psychiatric diagnosis decision trees applied in patient groups.

**Step 5**: Assess the accuracy of models.

ROC curve analyses were then applied to assess the efficacy of C&RT analysis results, using NSFT combined results as a biomarker/ the characteristic features for/of examined patient subgroups.

**Step 6**: Integration of data from C&RT analysis.

**Scheme 1:**
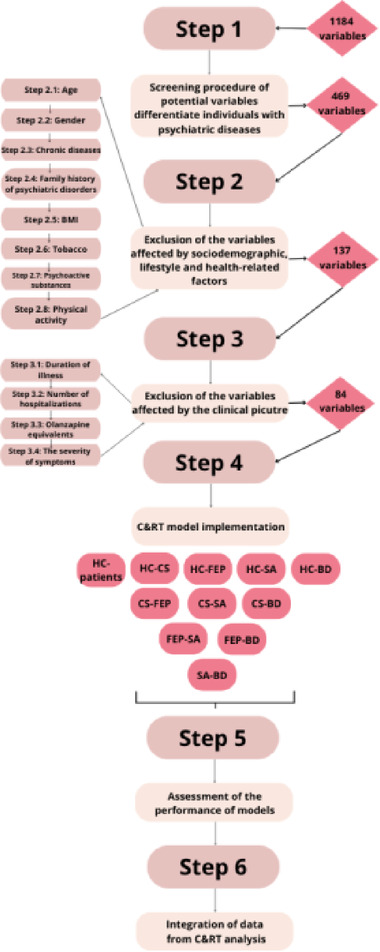
Graphical presentation of methodology for model fit.

We attempted to characterize individual mental disorders based on the obtained cut-off points of NSFT. Using this integrative approach (combining the results from obtained trees), we identified the variables that were repeated within two groups and removed them. The remaining variables were considered to be characteristics of particular groups of patients and HC. A summary of the following steps is presented in Scheme 1.

## Data Availability

The data presented in this study are available on request from the corresponding author.

## Abbreviations

NSFT: Niacin skin flush test
SCH: Schizophrenia
CS: Chronic schizophrenia
SA: Schizoaffective disorder
BD: Bipolar affective disorder
C&RT: Classification and regression tree
ROC: Receiver operating characteristic curve

## References

[1] Bosveld-van Haandel, L., Knegtering, R., Kluiter, H. & van den Bosch, R. J. Niacin skin flushing in schizophrenic and depressed patients and healthy controls. *Psychiatry Res.* **143**(2–3), 303–306. 10.1016/j.psychres.2005.10.010 (2006). 16837062 10.1016/j.psychres.2005.10.010

[2] Murrell, T. W. The cutaneous reaction to nicotinic acid (niacin)-Furfuryl. *Arch. Dermatol.* **79**(5), 545. 10.1001/archderm.1959.01560170043008 (1959). 10.1001/archderm.1959.01560170043008 13636446

[3] Hoffer, A. Adverse effects of niacin in emergent psychosis. *JAMA: J. Am. Med. Association*. **207**(7), 1355. 10.1001/jama.1969.03150200121025 (1969b). 4885414

[4] Fiedler, P., Wolkin, A. & Rotrosen, J. Niacin-induced flush as a measure of prostaglandin activity in alcoholics and schizophrenics. *Biol. Psychiatry*. **21**(13), 1347–1350. 10.1016/0006-3223(86)90321-5 (1986). 3756281 10.1016/0006-3223(86)90321-5

[5] Horrobin, D. F. Schizophrenia: A biochemical disorder? *Biomedicine* **32**(2), 54–55 (1980). PMID: 7388116. 7388116

[6] Wang, D. D. et al. Attenuated and delayed niacin skin flushing in schizophrenia and affective disorders: A potential clinical auxiliary diagnostic marker. *Schizophr Res.* **230**, 53–60 (2021). 33677199 10.1016/j.schres.2021.02.009

[7] Messamore, E. The niacin skin flush abnormality in schizophrenia: A quantitative dose–response study. *Schizophr Res.* **62**, 251–258 (2003). 12837522 10.1016/s0920-9964(02)00311-0

[8] Puri, B. K., Easton, T., Das, I., Kidane, L. & Richardson, A. J. The niacin skin flush test in schizophrenia: A replication study. *Int. J. Clin. Pract.* **55**, 368–370 (2001). 11501224

[9] Hudson, C. J., Lin, A., Cogan, S., Cashman, F. & Warsh, J. J. The niacin challenge test: Clinical manifestation of altered transmembrane signal transduction in schizophrenia? *Biol. Psychiatry*. **41**(5), 507–513. 10.1016/s0006-3223(96)00112-6 (1997). 9046982 10.1016/s0006-3223(96)00112-6

[10] Puri, B. K. & Singh, I. Normal phospholipid-related signal transduction in autism. *Prog. Neuropsychopharmacol. Biol. Psychiatry*. **26**(7–8), 1405–1407. 10.1016/s0278-5846(02)00303-2 (2002). 12502030 10.1016/s0278-5846(02)00303-2

[11] Katzman, M. et al. Methyl nicotinate-induced vasodilation in generalized social phobia. *Neuropsychopharmacology* **28**(10), 1846–1851. 10.1038/sj.npp.1300227 (2003). 12888773 10.1038/sj.npp.1300227

[12] Cyhlarova, E., Montgomery, P., Ross, M. A. & Richardson, A. J. Niacin skin test response in dyslexia. *Prostaglandins, Leukot. Essent. Fatty Acids*. **77**(2), 123–128. 10.1016/j.plefa.2007.08.005 (2007). 17890071 10.1016/j.plefa.2007.08.005

[13] Puri, B. K. Impaired phospholipid-related signal transduction in advanced Huntington’s disease. *Exp. Physiol.* **86**(5), 683–685. 10.1113/eph8602216 (2001). 11571498 10.1113/eph8602216

[14] Horrobin, D. F. Schizophrenia: A biochemical disorder? *Biomedicine* **32**, 54–55 (1980). 7388116

[15] Horrobin, D. F., Glen, A. I. M. & Vaddadi, K. The membrane hypothesis of schizophrenia. *Schizophr. Res.* **13**(3), 195–207. 10.1016/0920-9964(94)90043-4 (1994). 7841132 10.1016/0920-9964(94)90043-4

[16] Smesny, S. et al. Potential use of the topical niacin skin test in early psychosis -- a combined approach using optical reflection spectroscopy and a descriptive rating scale. *J. Psychiatr. Res.* **37**(3), 237–247. 10.1016/s0022-3956(03)00006-2 (2003). 10.1016/s0022-3956(03)00006-2 12650743

[17] Rybakowski, J. & Weterle, R. Niacin test in schizophrenia and affective illness. *Biol. Psychiatry*. **29**, 834–836 (1991). 2054456 10.1016/0006-3223(91)90202-w

[18] Glen, A. I. M. et al. Membrane fatty acids, niacin flushing and clinical parameters. *Prostaglandins Leukot. Essent. Fat. Acids*. **55**, 9–15 (1996). 10.1016/s0952-3278(96)90139-8 8888117

[19] Smesny, S. et al. Potential use of the topical niacin skin test in early psychosis—A combined approach using optical reflection spectroscopy and a descriptive rating scale. *J. Psychiatr Res.* **37**, 237–247 (2003). 12650743 10.1016/s0022-3956(03)00006-2

[20] Ross, B. Reduced vasodilatory response to methylnicotinate in schizophrenia as assessed by laser Doppler flowmetry. *Eur. Neuropsychopharmacol.* **14**, 191–197 (2004). 15056478 10.1016/S0924-977X(03)00145-7

[21] Liu, C. M. et al. Absent response to niacin skin patch is specific to schizophrenia and independent of smoking. *Psychiatry Res.* **152**(2–3), 181–187. 10.1016/j.psychres.2006.10.002 (2007). 17459487 10.1016/j.psychres.2006.10.002

[22] Smesny, S. et al. The influence of age and gender on niacin skin test results – implications for the use as a biochemical marker in schizophrenia. *J. Psychiatr. Res.* **38**(5), 537–543. 10.1016/j.jpsychires.2004.01.007 (2004). 15380405 10.1016/j.jpsychires.2004.01.007

[23] Smesny, S., Baur, K., Rudolph, N., Nenadic, I. & Sauer, H. Alterations of niacin skin sensitivity in recurrent unipolar depressive disorder. *J. Affect. Disord.* **124**(3), 335–340. 10.1016/j.jad.2009.12.017 (2010). 20116108 10.1016/j.jad.2009.12.017

[24] Kerr, M. et al. The topical niacin sensitivity test: An inter- and intra-rater reliability study in healthy controls. *Prostaglandins, Leukot. Essent. Fatty Acids*. **79**(1–2), 15–19. 10.1016/j.plefa.2008.06.001 (2008). 18656334 10.1016/j.plefa.2008.06.001

[25] Ward, P. E., Sutherland, J., Glen, E. M. T. & Glen, A. I. M. Niacin skin flush in schizophrenia: A preliminary report. *Schizophr. Res.* **29**(3), 269–274. 10.1016/s0920-9964(97)00100-x (1998). 9516668 10.1016/s0920-9964(97)00100-x

[26] Chang, S. S. et al. Impaired flush response to niacin skin patch among schizophrenia patients and their nonpsychotic relatives: The effect of genetic loading. *Schizophr. Bull.* **35**(1), 213–221. 10.1093/schbul/sbm153 (2008). 18203758 10.1093/schbul/sbm153 PMC2643969

[27] Wilson, D. W. S. & Douglass, A. B. Niacin skin flush is not diagnostic of schizophrenia. *Biol. Psychiatry*. **21**(10), 974–977. 10.1016/0006-3223(86)90274-x (1986). 3741914 10.1016/0006-3223(86)90274-x

[28] Stamatas, G. N. & Kollias, N. Blood stasis contributions to the perception of skin pigmentation. *J. Biomed. Opt.* **9**(2), 315. 10.1117/1.1647545 (2004a). 15065897 10.1117/1.1647545

[29] Xian, Y. L., Dai, Y., Gao, C. M. & Du, R. Dual-wavelength retinal images denoising algorithm for improving the accuracy of oxygen saturation calculation. *J. Biomed. Opt.* **22**(01), 1. 10.1117/1.jbo.22.1.016004 (2017). 10.1117/1.JBO.22.1.016004 28056145

[30] Gruensfelder, H. D. et al. Characterization of biological absorption spectra spanning the visible to the short-wave infrared. *J. Visualized Experiments*. **215**10.3791/67403 (2025). 10.3791/67403 PMC12061343 39878934

[31] Liu, P., Zhu, Z., Zeng, C. & Nie, G. Specific absorption spectra of hemoglobin at different PO2 levels: Potential noninvasive method to detect PO2 in tissues. *J. Biomed. Opt.* **17**(12), 125002. 10.1117/1.jbo.17.12.125002 (2012). 23208210 10.1117/1.JBO.17.12.125002

[32] Burns, J. M., Saager, R., Majaron, B., Jia, W. & Anvari, B. Optical properties of biomimetic probes engineered from erythrocytes. *Nanotechnology* **28**(3), 035101. 10.1088/1361-6528/28/3/035101 (2016). 27966473 10.1088/1361-6528/28/3/035101 PMC5189990

[33] Zijlstra, W. G., Buursma, A. & van Assendelft, O. W. *Visible and near Infrared Absorption Spectra of Human and Animal Haemoglobin*. 10.1201/9780429071096 (2021).

[34] Yang, B. et al. Color structured light imaging of skin. *J. Biomed. Opt.* **21**(5), 050503. 10.1117/1.jbo.21.5.050503 (2016). 27207112 10.1117/1.JBO.21.5.050503 PMC4890357

[35] Cai, L. & Pfob, A. *Processing HSV Colored Medical Images and Adapting Color Thresholds for Computational Image Analysis: a Practical Introduction to an open-source tool*. https://arxiv.org/abs/2404.17878 (2024).

[36] Kumar, K. & chaduvula A Detailed Survey On Feature Extraction Techniques In Image Processing For Medical Image Analysis. (2021).

[37] Hassan, R., Ema, R. & Islam, T. Color Image Segmentation using Automated K-Means Clustering with RGB and HSV Color Spaces. **17**. 33–41. (2017).

[38] Hudson, C., Gotowiec, A., Seeman, M., Warsh, J. & Ross, B. M. Clinical subtyping reveals significant differences in calcium-dependent phospholipase A2 activity in schizophrenia. *Biol. Psychiatry*. **46**, 401–405 (1999). 10435206 10.1016/s0006-3223(99)00010-4

[39] Puri, B. K., Hirsch, S. R., Easton, T. & Richardson, A. J. A volumetric biochemical niacin flush-based index that noninvasively detects fatty acid deficiency in schizophrenia. *Prog Neuro-Psychopharmacol Biol. Psychiatry*. **26**, 49–52 (2002). 10.1016/s0278-5846(01)00220-2 11853118

[40] Tavares, H., Yacubian, J., Talib, L. L., Barbosa, N. R. & Gattaz, W. F. Increased phospholipase A2 activity in schizophrenia with absent response to niacin. *Schizophr Res.* **61**, 1–6 (2002). 10.1016/s0920-9964(02)00281-5 12648730

[41] Lin, S. H. et al. Familial aggregation in skin flush response to niacin patch among schizophrenic patients and their nonpsychotic relatives. *Schizophr Bull.* **33**, 174–182 (2006). 16936284 10.1093/schbul/sbl038 PMC2632299

[42] Yao, J. K. et al. Prevalence and specificity of the abnormal niacin response: A potential endophenotype marker in schizophrenia. *Schizophr Bull.* **42**, 369–376 (2015). 26371338 10.1093/schbul/sbv130 PMC4753599

[43] Sun, L. et al. Identification of the niacin-blunted subgroup of schizophrenia patients from mood disorders and healthy individuals in Chinese population. *Schizophr Bull.* **44**, 896–907 (2017). 10.1093/schbul/sbx150 PMC6007359 29077970

[44] Karakula-Juchnowicz, H. et al. SKINREMS—A new method for assessment of the niacin skin flush test response in schizophrenia. *J. Clin. Med.* **9**, 1848 (2020). 32545806 10.3390/jcm9061848 PMC7356909

[45] Hu, Y. et al. A potential objective marker in first-episode schizophrenia based on abnormal niacin response. *Schizophr Res.* **243**, 405–412 (2021). 34187733 10.1016/j.schres.2021.06.028

[46] Zhang, T. et al. Association of attenuated niacin response with inflammatory imbalance and prediction of conversion to psychosis from clinical high-risk stage. *J. Clin. Psychiatry*, **84**(5). (2023). 10.4088/JCP.22m14731 37471530

[47] Ju, M. et al. Cognitive impairments in first-episode psychosis patients with attenuated niacin response. *Schizophr. Res.* **40**, 100346 (2025). 10.1016/j.scog.2025.100346 PMC11803152 39925786

[48] Wang, J. et al. Efficacy of identifying treatment-resistant and non-treatment-resistant schizophrenia using niacin skin flushing response combined with clinical feature. *Schizophrenia*. **11**(1) (2025). 10.1038/s41537-025-00567-4 PMC11806114 39920210

[49] Lyu, X., Goperma, R., Wang, D., Wan, C. & Zhao, L. An open dataset and machine learning algorithms for niacin skin-flushing response based screening of psychiatric disorders. *BMC Psychiatry*. **25**(1). (2025). 10.1186/s12888-025-07196-2 PMC12323195 40760690

[50] Chen, H. et al. Blunted niacin skin flushing response in violent offenders with schizophrenia: A potential auxiliary diagnostic biomarker. *J. Psychiatr. Res.* **184**, 249–255 (2025). 40058163 10.1016/j.jpsychires.2025.02.059

[51] Li, L. Application of machine learning and data mining in medicine: Opportunities and considerations. *Artif. Intell.*10.5772/intechopen.113286 (2023).

[52] Leucht, S., Samara, M., Heres, S. & Davis, J. M. Dose equivalents for antipsychotic drugs: The DDD method: Table 1. *Schizophr. Bull.* **42**(suppl 1). 10.1093/schbul/sbv167 (2016). 10.1093/schbul/sbv167 PMC4960429 27460622

[53] Sitarz, R. et al. Niacin skin flush backs—from the roots of the test to nowadays hope. *J. Clin. Med.* **12**(5), 1879. 10.3390/jcm12051879 (2023). 36902666 10.3390/jcm12051879 PMC10003235

